# The E3 Ubiquitin Ligase TRIM65 Negatively Regulates Inflammasome Activation Through Promoting Ubiquitination of NLRP3

**DOI:** 10.3389/fimmu.2021.741839

**Published:** 2021-08-26

**Authors:** Tiantian Tang, Ping Li, Xinhui Zhou, Rui Wang, Xiuqin Fan, Mengyi Yang, Kemin Qi

**Affiliations:** Laboratory of Nutrition and Development, Key Laboratory of Major Diseases in Children, Ministry of Education, Beijing Pediatric Research Institute, Beijing Children's Hospital, Capital Medical University, National Center for Children's Health, Beijing, China

**Keywords:** NLRP3 inflammasome, TRIM65, ubiquitination, posttranslational regulation, inflammasome-driven diseases

## Abstract

The dysregulation of NLRP3 inflammasome plays a critical role in pathogenesis of various human inflammatory diseases, thus NLRP3 inflammasome activation must be tightly controlled at multiple levels. However, the underlying mechanism regulating NLRP3 inflammasome activation remains unclear. Herein, the effects of Tripartite motif-containing protein 65 (TRIM65) on NLRP3 inflammasome activation and the underlying molecular mechanism were investigated *in vitro* and *in vivo*. Inhibition or deletion of *Trim65* could significantly strengthen agonist induced NLRP3 inflammasome activation in THP-1 cells and BMDMs, indicated by increased caspase-1 activation and interleukin-1β secretion. However, TRIM65 had no effect on poly (dA: dT)-induced AIM2 inflammasome activation or flagellin-induced IPAF inflammasome activation. Mechanistically, immunoprecipitation assays demonstrated that TRIM65 binds to NACHT domain of NLRP3, promotes lys48- and lys63- linked ubiquitination of NLRP3 and restrains the NEK7-NLRP3 interaction, thereby inhibiting NLRP3 inflammasome assembly, caspase-1 activation, and IL-1β secretion. *In vivo*, three models of inflammatory diseases were used to confirm the suppression role of TRIM65 in NLRP3 inflammasome activation. TRIM65-deficient mice had a higher production of IL-1β induced by lipopolysaccharide in sera, and more IL-1β secretion and neutrophil migration in the ascites, and more severity of joint swelling and associated IL-1β production induced by monosodium urate, suggesting that TRIM65 deficiency was susceptible to inflammation. Therefore, the data elucidate a TRIM65-dependent negative regulation mechanism of NLRP3 inflammasome activation and provide potential therapeutic strategies for the treatment of NLRP3 inflammasome-related diseases.

## Introduction

The NLRP3 inflammasome, a multimeric protein complex composed of the NOD-like receptor (NLR) family member NLRP3, adaptor ASC and downstream effector pro-caspase-1, plays a key role in host defense against pathogen infection and endogenous “danger signals” (1–3). Activation of the NLRP3 inflammasome triggers the self-cleavage of pro-caspase-1, resulting in downstream maturation and release of the inflammatory cytokines interleukin-1β (IL-1β) and IL-18, which further expand inflammation (4). Numerous studies have shown that excessive activation of the NLRP3 inflammasome causes multiple autoinflammatory and autoimmune-associated metabolic disorders, including gout, type 2 diabetes, Alzheimer’s disease, atherosclerosis and cryopyrin-associated periodic syndromes (CAPS) (5–8). Thus, the balance between positive and negative regulation of the NLRP3 inflammasome is critical during the immune response, and activation of the NLRP3 inflammasome must be tightly controlled and regulated at multiple levels. However, the regulatory networks that control NLRP3 inflammasome activation, especially endogenous negative regulatory mechanisms, remain elusive.

Recently, a series of studies have demonstrated that activation of the NLRP3 inflammasome can be tightly controlled and regulated through posttranslational modifications (PTMs) of NLRP3, including phosphorylation (9), ubiquitination (10, 11), sumoylation (12), alkylation (13), and S-nitrosylation (14). Among them, ubiquitination, as one of the most versatile posttranslational modifications, play an integral role in the control of the NLRP3 inflammasome activity. Multiple E3 ubiquitin ligases including FBXL2 (15), TRIM31 (16), MARCH7 (17), ARIH2 (10), RNF125, Cbl-b (18), etc., have been reported as endogenous negative regulators of NLRP3 inflammasome activation. FBXL2, TRIM31 and Cbl-b cause K48-linked ubiquitination and proteasomal degradation of NLRP3, reducing inflammasome activation; MARCH7 promotes K48-linked ubiquitination and autophagic degradation of NLRP3; RNF125 induces K63-linked ubiquitination of NLRP3; ARIH2 was required for NLRP3 ubiquitination linked through K48 and K63. Moreover, the reduction of FBXL2 and increase of TRIM31 could be induced by lipopolysaccharide (LPS) to strengthen stability of the NLRP3 protein (10, 15–18). Therefore, the effects of these E3 ubiquitin ligases on NLRP3 inflammasome activation could be attributable to three different types of mechanisms (lys48-, lys63- or mixed lys48- and lys63- linked ubiquitination) and two different degradation pathways (autophagic and proteasomal degradation). However, the E3 ubiquitin ligases responsible for ubiquitinating NLRP3 are not fully characterized and the function of mixed ubiquitination on NLRP3 inflammasome activity remains largely unclear.

TRIM65, a number of the tripartite motif (TRIM) family, has E3 ubiquitin ligase activity and plays a crucial role in tumor progression (19, 20) and miRNA pathway (21), while the function in innate immunity is still unclear. Previous research showed that TRIM65 plays an important role in MDA5-mediated antiviral innate immunity and limitation of lung inflammation (22–24). However, the biological function of TRIM65 in inflammasome-associated immune response remains little known.

Here, this study demonstrate that the E3 ubiquitin ligase TRIM65 is an endogenous negative regulator of NLRP3 inflammasome activation. TRIM65 was found to directly bind NLRP3 and promotes its mixed lys48- and lys63- linked ubiquitination in macrophages. NLRP3 agonists induced TRIM65 reduction to favor NLRP3 inflammasome assembly and activation. Thus, TRIM65 deficiency impairs NLRP3 ubiquitination and enhances NLRP3 inflammasome activation, but has no effects on AIM2 or IPAF inflammasome activation. Consistent with that, TRIM65 deficiency aggravated LPS-induced systemic inflammation and MSU-induced peritonitis and gouty arthritis *in vivo*. Thus, TRIM65 might be a potential therapeutic target for NLRP3-dependent inflammatory diseases.

## Materials and Methods

### Mice

*Trim65*^−/−^ mice were donated by Professor Rongbin Zhou and Professor Wei Jiang of the University of Science and Technology of China (USTC). All animal experiments were approved by the Ethics Committee of the University of Science and Technology of China. *Wild-type* C57BL/6 mice were purchased from Beijing HFK Bioscience Co., Ltd. All mice were on a C57BL/6 background and fed in a SPF (specific pathogen-free) facility at the University of Science and Technology of China and the Chinese Center for Disease Control and Prevention.

### Reagents

Phorbol-12-myristate-13-acetate (PMA), LPS, Pam3CSK4, MSU, ATP, nigericin, flagellin, MG132, 3-MA and poly (dA:dT) were purchased from Sigma-Aldrich (St. Louis, MO, USA). DAPI, M-MLV and Lipofectamine 2000 were purchased from Thermo Scientific (CA, USA). Pam3CSK4 (tripalmitoyl cysteinyl seryl tetralysine lipopeptide), ultrapure LPS and R837 were obtained from InvivoGen (San Diego, CA, USA). SYRB Green and TRIzol reagent were obtained from TAKARA (Tokyo, Japan). Polyethylenimine was supplied by Polysciences (PA, USA), and protein G agarose was purchased from Millipore (MA, USA). DOTAP was purchased from Sigma-Aldrich (St. Louis, MO, USA) and Applygen (Beijing, China). Anti-Flag agarose beads and anti-Flag (M2) were purchased from Sigma-Aldrich (St. Louis, MO, USA). Anti-GFP and anti–β-actin antibodies were purchased from Abmart (Shanghai, China). Anti-ubiquitin and anti–human TRIM65 antibodies were purchased from Santa Cruz Biotechnology (Santa Cruz, CA, USA). Anti-human cleaved IL-1β, anti-human caspase-1, anti-lys48-linked polyubiquitin and anti-lys63-linked polyubiquitin antibodies were obtained from Cell Signaling Technology (Beverly, MA, USA). Anti-mouse caspase-1 (p20) and anti-NLRP3 antibodies were obtained from AdipoGen Life Sciences (San Diego, CA, USA). Anti-mouse IL-1β antibody was obtained from R&D Systems (MPLS, USA). Anti-human pro-IL-1β and anti-HA antibodies were obtained from Proteintech (Wuhan, Hubei, China). ShRNA targeting TRIM65 was purchased from Sigma-Aldrich (St. Louis, MO, USA). The ELISA Kit of mouse IL-1β, mouse IL-6 and mouse TNF-α were from R&D Systems (MPLS, USA). The ELISA Kit of human IL-1β was obtained from ExCell Biotech (Taicang, Jiangsu, China). The HEK-293T, THP-1 and L929 cell lines (which were shown to be negative for mycoplasma contamination) were obtained from American Type Culture Collection (ATCC).

### Cell Preparation and Stimulation

HEK-293T and L929 cells were cultured in DMEM, and human THP-1 cells were grown in RPMI 1640 medium. Both culture media contained 10% fetal bovine serum (FBS) and 1% penicillin-streptomycin. BMDMs were isolated and cultured as described (25). To stimulate the NLRP3 inflammasome, 5×10^5^/ml differentiated THP-1 cells (100 nM PMA, 3 h) or BMDMs were plated in 12-well or 24-well plates. After 12–18 h, the supernatant was replaced with medium containing LPS (50 ng/ml) or Pam3CSK4 (400 ng/ml, TLR2 ligand, for noncanonical inflammasome activation) and incubated for 3-4 h. Then, the cells were stimulated with MSU or R837 for 4 h, with ATP or nigericin for 30 min, or transfected with poly (dA:dT) (0.5 μg/ml, 4 h) or LPS (500 ng/ml, 16 h) by used Lipofectamine 2000, or flagellin (200 ng/ml, 4 h) by used DOTAP. Specifically, the cells were treated with different doses of proteasome inhibitors (MG132, 1 h) or autophagy inhibitors (3-MA, 3 h) before stimulated with agonist R837 in **Figure 4C**. Cell lysates and supernatants were analyzed *via* immunoblotting or ELISA.

### ELISA

Supernatants from cell culture, tissue culture, serum or peritoneal lavage fluid were assayed for mouse IL-1β (R&D, DY401, 15.6-1,000 pg/ml), mouse IL-6 (R&D, DY406-05, 15.6-1,000 pg/ml), mouse TNF-α (R&D, DY410-05, 31.2-2,000 pg/mL) and human IL-1β (ExCell Biotech, EH001, 4-500 pg/ml) according to the manufacturer’s instructions.

### Generation of THP-1 Cells Expressing shRNA

The protocol used to generate THP-1 cells stably expressing shRNA specific for TRIM65 has been described previously (25). Sequences of shRNA used in this paper are as follows: shTRIM65 (1), 5′-GAATTATCGCAATCTGACCTT-3′; shTRIM65 (2), 5′-CCGTCCTGTCTTGTAGTCTTT-3′; shTRIM65 (3), 5′-TCTGGCAGAATTATCGCAATC-3′.

### Confocal Microscopy

Plasmids were transfected into HEK-293T cells, which were plated on coverslips the day before transfection with the transfection reagent polyethylenimine. After 24 h, the cells were treated as described previously (22). A Zeiss LSM 700 microscope was used for confocal microscopy analysis and LSM Image Browser was used for image analysis.

### Transfection and Immunoprecipitation

HEK-293T cells were plated in 6-well plates overnight, and plasmids were transfected *via* polyethylenimine. After 24 h, the cells were processed as previously reported (22). THP-1 and LPS-primed BMDMs (1 × 10^7^) were stimulated with nigericin (0.5 h), then the cells were processed as previously reported (26). Extracts were immune- precipitated by incubation with anti-Flag beads or anti-IgG beads for 3 h at 4°C. The extracts with incubated with control IgG, anti-VSV antibody, anti-NLRP3 antibody, or anti-TRIM65 antibody and protein A/G agarose resin for 12 h at 4°C. Then, the antibody-bead mixtures were washed in NP-40 lysis buffer (four times) and analyzed by immunoblotting.

### LPS-Induced Systemic Inflammation *In Vivo*

*Trim65^+/+^* or *Trim65^-/-^* mice (8-10 weeks) were injected intraperitoneally with LPS (20 mg/kg body weight). After 4 h, mice were killed, and serum IL-1β were measured by ELISA (17).

### MSU-Induced Peritonitis and Gouty Arthritis *In Vivo*

*Trim65^+/+^* or *Trim65^-/-^* mice (8-10 weeks) were injected intraperitoneally with MSU crystals (1 mg, dissolved in 0.5 ml sterile PBS). After 6 h, peritoneal cavities were washed with 10 ml cold PBS. Peritoneal lavage fluid was assessed by flow cytometry (BD FACS caliber), and the recruitment of polymorphonuclear neutrophils was analyzed by neutrophil markers Gr-1 and CD11b. The IL-1β level in peritoneal lavage fluid was determined by ELISA (27).

*Trim65^+/+^* or *Trim65^-/-^* mice (8-10 weeks) were injected intraarticularly with MSU crystals (0.5 mg, dissolved in 20 μl sterile PBS). Then, the size of joints was measured at different time points. After 24 h, the patella was photographed, isolated and cultured in 200 μl OPTI-MEM medium containing 1% Penicillin-Streptomycin at room temperature for 1 h. Finally, the level of IL-1β in medium was determined by ELISA (28).

### Statistical Analysis

Data was presented as the mean ± SEM. Statistical analyses of two groups were performed with the unpaired Student’s *t-test* (GraphPad Prime 8.0 software). No data points were excluded. The researchers were not blinded during sample collection or data analysis. Sample sizes were selected on the basis of preliminary results to ensure adequate data for statistical analyses. All statistical tests were 2-sided, and differences with a *P*-value < 0.05 were considered statistically significant.

## Results

### TRIM65 Interacts With NLRP3

**Figure 1:**
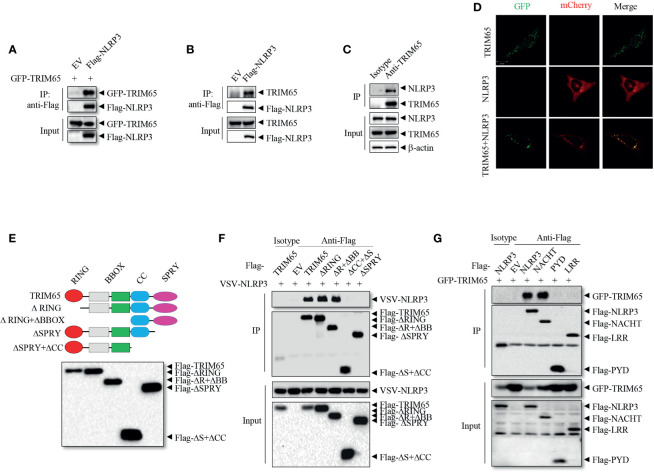
TRIM65 interacts with NLRP3. **(A)** Flag-NLRP3 was cotransfected with GFP-TRIM65 in HEK-293T cells. The interaction between NLRP3 and TRIM65 was analyzed by immunoprecipitation and western blotting. **(B)** Flag-NLRP3 was transfected and expressed in HEK-293T cells. The interaction between NLRP3 and endogenous TRIM65 was assayed by immunoprecipitation and western blotting. **(C)** The lysates of PMA-differentiated and LPS-primed THP-1 cells were immunoprecipitated with anti-NLRP3 antibody, and the interaction between endogenous NLRP3 and endogenous TRIM65 was analyzed by western blotting. **(D)** mCherry-NLRP3 was cotransfected with GFP-TRIM65 in HEK-293T cells. The colocalization of NLRP3 and TRIM65 was assayed by confocal microscopy. Bars: (white) 20 mm. **(E)** Diagram of full-length TRIM65 and TRIM65 deletion mutants (ΔRING, ΔR+ΔBB, ΔCC+ΔS or ΔSPRY). **(F)** Flag-TRIM65 and TRIM65 deletion mutants were individually cotransfected with VSV-NLRP3 in HEK-293T cells. The interactions between NLRP3 and TRIM65 or its different domains were analyzed by immunoprecipitation and western blotting. **(G)** Full-length or truncated Flag-NLRP3 (Flag-PYD, Flag-NACHT or Flag-LRR) was cotransfected with GFP-TRIM65 in HEK-293T cells. The interaction between TRIM65 and full-length or truncated NLRP3 was analyzed by immunoprecipitation and western blotting. Data are representative of three independent experiments. EV, empty vector; Input, cell extract without immunoprecipitation.

To investigate the mechanisms of NLRP3 inflammasome activation, liquid chromatography-mass spectrometry (LC-MS) was used to screen proteins that interact with NLRP3 (21); the results showed that among peptides in the precipitate, the E3 ligase TRIM65 was one of the best matches. Next the interaction between TRIM65 and NLRP3 was confirmed. Specifically, an *in vitro* immunoprecipitation (IP) assay demonstrated that overexpressed NLRP3 could directly interact with both exogenous and endogenous TRIM65 in human embryonic kidney 293T (HEK-293T) cells (**Figures 1A, B**). Further in PMA-differentiated THP-1 cells, there was a significant endogenous interaction between them (**Figure 1C**). Consistently, confocal microscopy analysis showed the colocalization of TRIM65 and NLRP3 in 293T cells (**Figure 1D**). These data demonstrated that TRIM65 could directly interact with NLRP3. Considering that NLRP3 is composed of three separate domains (an amino-terminal pyrin domain, a central NACHT, and a C-terminal LRR) (29), and TRIM65 contains four domains (RING, BBOX, Coil-Coil and SPRY) (22), a series of GFP-tagged truncated TRIM65 mutants were constructed and expressed in HEK-293T cells to localize the domains of TRIM65 and NLRP3 and their interaction (**Figure 1E**). Immunoprecipitation assay showed that NLRP3 interacted with full-length TRIM65, RING domain deletion mutant (ΔRING) and RING-BBOX domain deletion mutant (ΔR+ΔBB), but did not interact with the SPRY domain deletion mutant (ΔSPRY) or SPRY-CC domain deletion mutant (ΔCC+ΔS) (**Figure 1F**), indicating that TRIM65 interacts with NLRP3 *via* its SPRY domain. At the same time, NACHT domain has been demonstrated to be the exact domain of NLRP3 interacting with TRIM65, as opposed to the PYD or LRR domain of NLRP3 (**Figure 1G**). It is reported that, besides NLRP3, other Pattern Recognition Receptors (PRRs, such as NLRP1, IPAF and AIM2) are also expressed and form specific inflammasomes in macrophages. Thus, interactions of TRIM65 with these PRRs were explored, and the results showed that TRIM65 could interact with NLRP1 and IPAF but not with AIM2 (**Figure S1**). Collectively, these findings illustrate that the SPRY domain of TRIM65 can directly target NLRP3 by binding its NACHT domain.

### TRIM65 Inhibits NLRP3 Inflammasome Activation in Macrophages

**Figure 2:**
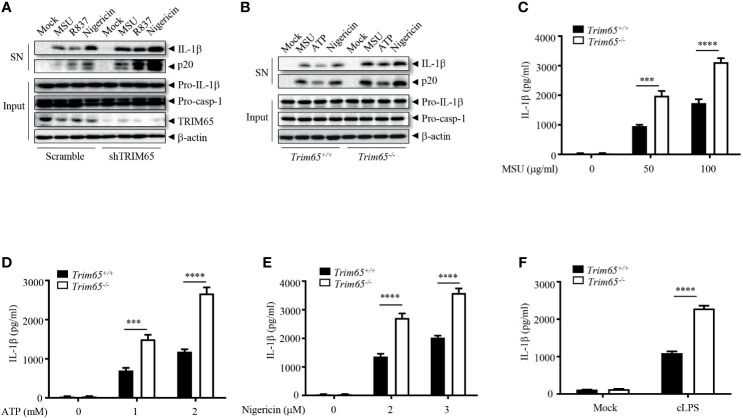
TRIM65 inhibits NLRP3 inflammasome activation in macrophage. **(A)** PMA-differentiated and LPS-primed THP-1 cells stably expressing shRNA targeting TRIM65 mRNA were stimulated with MSU, R837 or nigericin. Cleaved IL-1β, activated caspase-1 (P20) in the SN and pro-IL-1β and pro-caspase-1 in the input were analyzed by western blotting. **(B-F)** LPS-primed BMDMs from *wild-type* and *Trim65^-/-^* mice were stimulated with MSU, ATP, nigericin **(B–E)**, or cytosolic LPS **(F)**. Cleaved IL-1β, activated caspase-1 (P20) in the culture supernatants and pro-IL-1β and pro-caspase-1 in cell lysates were analyzed by western blotting **(B)**. Cleaved IL-1β in the culture supernatants was assayed by ELISA **(C–F)**. Data are representative of three independent experiments of duplicate biological repeats **(A, B)**. SN, media supernatants; Input, cell extracts. Student’s *t*-test, ***P < 0.001, ****P < 0.0001. Data are shown as the means ± SEM of three independent experiments **(C–F)**.

To investigate whether the observed interaction between TRIM65 and NLRP3 is important for the activation of the NLRP3 inflammasome, THP-1 cells stably expressing shRNA targeting TRIM65 to suppress the expression of endogenous TRIM65 expression were established (**Figures 2A** and **S2A**). Inhibition of *trim65* expression significantly strengthened monosodium urate (MSU), nigericin, or R837 (also known as imiquimod, one of the TLR7 ligands)-induced NLRP3-dependent caspase-1 activation and IL-1β maturation but did not change LPS (TLR4 ligand)-induced pro-IL-1β expression (**Figures 2A** and **S2B**). These results suggest that TRIM65 could inhibit the activation of the NLRP3 inflammasome in response to multiple agonists. To confirm the function of TRIM65 in NLRP3 inflammasome activation, bone marrow-derived macrophages (BMDMs) from *Trim65^-/-^* mice, obtained through transcription activator–like effector nuclease (TALEN) technology (22), were used to repeat the above experiments. The results showed that *Trim65* deletion could increase levels of caspase-1 activation and mature IL-1β secretion upon NLRP3 stimulation with MSU, ATP or nigericin (**Figure 2B**). Meanwhile, the ELISA analysis of mature IL-1β secreted into the supernatant (SN) after different dose treatments of NLRP3 agonists further confirmed the immunoblotting results (**Figures 2C–E**). In addition, TRIM65 had no effect on LPS-induced pro-IL-1β (**Figure S3A**) and NLRP3 expression or TNF-α and IL-6 secretion (**Figures S3B, C**), indicating that TRIM65 inhibits IL-1β production by affecting NLRP3 inflammasome activation instead of priming signaling. In addition to canonical agonists such as MSU, ATP and nigericin, NLRP3 inflammasome can also be activated by cytosolic LPS (cLPS) produced by gramnegative bacteria, which induce caspase-11-dependent noncanonical NLRP3 inflammasome activation (30). Then, whether TRIM65 affects cLPS induced noncanonical NLRP3 inflammasome activation were tested. The result showed that TRIM65 knockout significantly advanced mature IL-1β production and noncanonical NLRP3 inflammasome activation induced by cLPS in BMDMs (**Figure 2F**). TRIM65 had no effect on poly (dA: dT)-induced AIM2 inflammasome activation (**Figures S4A–C**) or flagellin (TLR5 ligand)-induced IPAF inflammasome activation (**Figures S4D, E**), which suggested that specific role for TRIM65 in inhibition of the NLRP3 inflammasome activation. Overall, these results indicate that TRIM65 specifically inhibits canonical and noncanonical NLRP3 inflammasome activation in macrophages.

### TRIM65 Promotes NLRP3 lys48- and lys63- Linked Ubiquitination

**Figure 3:**
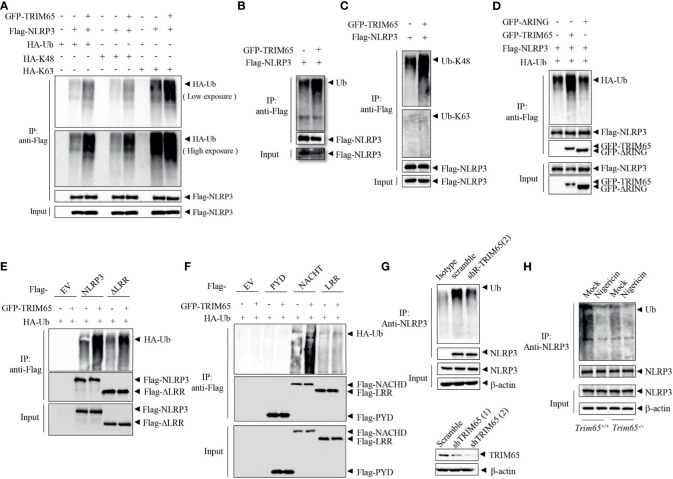
TRIM65 promotes NLRP3 lys48- and lys63- linked ubiquitination. **(A)** Flag-NLRP3 and GFP-TRIM65 were cotransfected with HA-Ubiquitin, HA-Ubiquitin (K48-linked only), or HA-Ubiquitin (K63-linked only) in HEK-293T cells. The ubiquitination of NLRP3 was analyzed by immunoprecipitation and western blotting. **(B, C)** Flag-NLRP3 was cotransfected with GFP-TRIM65 in HEK- 293T cells. The overall ubiquitination **(B)**, K48-linked ubiquitination **(C)** and K63-linked ubiquitination **(C)** of NLRP3 were analyzed by immunoprecipitation and western blotting. **(D)** Flag-NLRP3 was cotransfected with GFP-TRIM65 or GFP-TRIM65 lacking the RING domain in HEK-293T cells. The ubiquitination of NLRP3 was analyzed by immunoprecipitation and western blotting. **(E, F)** Full-length Flag-NLRP3 and NLRP3 truncation mutants were individually cotransfected with GFP-TRIM65 in HEK-293T cells. Ubiquitination of NLRP3 or its truncation mutants was analyzed by immunoprecipitation and western blotting. **(G)** Endogenous ubiquitination of NLRP3 in PMA-differentiated and LPS-primed THP-1 cells stably expressing scrambled shRNA or shRNA targeting TRIM65 mRNA was analyzed by immunoprecipitation and western blotting. **(H)** LPS-primed BMDMs from *wild-type* or *Trim65^-/-^* mice were stimulated with nigericin, and the endogenous ubiquitination of NLRP3 was analyzed by immunoprecipitation and western blotting. Data are representative of two or three independent experiments. EV, empty vector.

Next, Mechanisms by which TRIM65 suppressed NLRP3 inflammasome activation were studied. As an E3 ubiquitin ligase, does TRIM65 promote NLRP3 ubiquitination and restrain NLRP3 activation inflammasome *via* its ubiquitin ligase function? Firstly, TRIM65, NLRP3 and HA-ubiquitin were transfected into HEK-293T cells, and an immunoprecipitation (IP) assay showed that overexpression of TRIM65 significantly promoted NLRP3 ubiquitination (**Figure 3A**). Previous study showed that NLRP3 undergoes both lys48- and lys63- linked ubiquitination, which mediates negative regulation of NLRP3 inflammasome activation (10, 31). Then, two types of NLRP3 ubiquitination, lys48- and lys63- linked ubiquitination induced by TRIM65 were found *via* IP assay. It was worth noting that TRIM65 was more efficient in mediating Lys63- than Lys48- linked ubiquitination (**Figure 3A**). To verify this result, the TRIM65-induced ubiquitination of NLRP3 with endogenous ubiquitin was assessed, which yielded the same result obtained with exogenous ubiquitin (**Figures 3B, C**), indicating that TRIM65 could induce both lys48- and lys63- linked ubiquitination of NLRP3. In order to further determine the key domain of TRIM65 for ubiquitination, Flag-tagged full-length NLRP3 with GFP-tagged TRIM65 in which the RING domain had been deleted (ΔRING) were cotransfected and the results indicated that TRIM65 without its RING domain (ΔRING) could not induce the ubiquitination of NLRP3, suggesting that the TRIM65-promoted ubiquitination of NLRP3 is RING domain dependent (**Figure 3D**). Next, the structural domains of NLRP3 involved in NLRP3 ubiquitination were determined. Full-length GFP-tagged TRIM65 was individually coexpressed with Flag-tagged NLRP3 and four truncated NLRP3 proteins. IP assays showed that TRIM65 induced ubiquitination of NACHT domain or the truncated proteins lacking the LRR domain (ΔLRR), and did not induce ubiquitination of LRR domain or PYD domain (**Figures 3E, F**). These results suggested that the NACHT domain of NLRP3 could be ubiquitinated by the RING domain of TRIM65.

Juliana et al. demonstrated that NLRP3 undergoes basal ubiquitination and is activated by a two-independent-step deubiquitination mechanism initiated by the priming signal and further potentiated by the NLRP3 inflammasome agonist (26). To determine whether the E3 ubiquitin ligase activity of TRIM65 plays a critical role in NLRP3 ubiquitination and further affects NLRP3 inflammasome activation, the change in NLRP3 ubiquitination in THP-1 cells were analyzed. NLRP3 was ubiquitinated in both resting and priming macrophages, but nigricin stimulation could reduce the ubiquitination of NLRP3 (**Figure S5**). Subsequently, the ubiquitination of NLRP3 were tested in THP-1 cells which stably expressing shRNA targeting TRIM65. As shown in **Figure 3G**, the expression of TRIM65 was silenced by shRNA in THP-1 cells, and the second shRNA with better knockdown effect was selected for the following IP experiment. Compared with wild type, ubiquitination of NLRP3 was attenuated in TRIM65-konckdown THP-1 cells. Furthermore, to confirm the function of TRIM65 in response to an agonist, the ubiquitination of NLRP3 stimulated with the corresponding agonist was determined in *wild-type* and *Trim65^-/^* ^-^ BMDMs. Consistently, the ubiquitination of NLRP3 in TRIM65-deficient macrophages was suppressed and significantly reduced upon stimulation with the agonist nigericin (**Figure 3H**). Thus, NLRP3 ubiquitination was impaired in TRIM65-deficient cells, and agonist stimulation further decreased its ubiquitination. Above all, these results indicate that TRIM65 promote lys48- and lys63- linked NLRP3 ubiquitination by its E3 ubiquitin ligase activity, which is critical for the inhibition of NLRP3 inflammasome activation in resting macrophages.

### NLRP3 Agonists Facilitate TRIM65 Reduction to Favor NEK7-NLRP3 Interaction and NLRP3 Inflammasome Assembly

**Figure 4:**
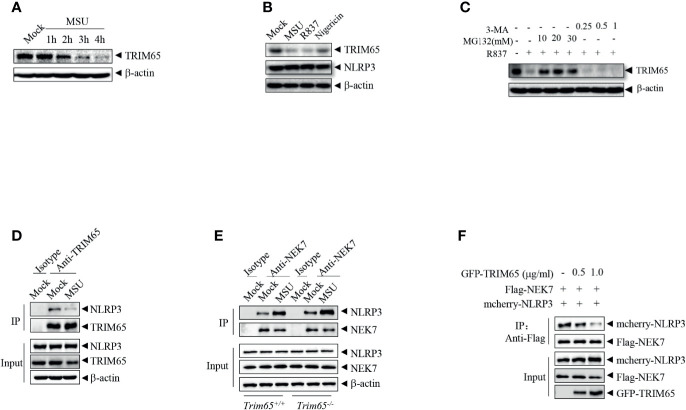
NLRP3 agonists promote TRIM65 reduction to favor NEK7-NLRP3 interaction and NLRP3 inflammasome assembly. **(A)** PMA-differentiated and LPS-primed THP-1 cells were stimulated with MSU for different durations, and the expression of TRIM65 was analyzed by western blotting. **(B)** PMA-differentiated and LPS-primed THP-1 cells were stimulated with MSU, R837 or nigericin, and the expression of TRIM65 was analyzed by western blotting. **(C)** PMA-differentiated and LPS-primed THP-1 cells were treated with different doses of proteasome inhibitors (MG132, 1 h) or autophagy inhibitors (3-MA, 3 h), and then stimulated with R837 for 4 h, and the expression of TRIM65 was analyzed by western blotting. **(D)** PMA-differentiated and LPS-primed THP-1 cells were stimulated with MSU, and the endogenous interaction between NLRP3 and TRIM65 was analyzed by immunoprecipitation and western blotting. **(E)** LPS-primed BMDM cells were stimulated with ATP and Nigericin, and the interaction between NLRP3 and NEK7 was analyzed by immunoprecipitation and western blotting. **(F)** Mcherry-NLRP3 and Flag-NEK7 were cotransfected with or without GFP- TRIM65 in HEK-293T cells. The interaction between NLRP3 and NEK7 was analyzed by immunoprecipitation and western blotting. Data are representative of three independent experiments.

In order to further exploration of the function and mechanisms of TRIM65 in NLRP3 activation, the expression of TRIM65 in NLRP3 activation stimulated by agonist were examined. As shown in **Figure 4A**, we found that treatment with the NLRP3 agonist MSU could reduce levels of TRIM65 in a time dependent manner in PMA-differentiated and LPS-priming THP-1 cells. NLRP3 agonist-induced the reduction of TRIM65 was not specific to only MSU but also the other agonists R837 and nigericin (**Figure 4B**). Then, whether proteasome or autophagy mediated the degradation of TRIM65? Our results showed that proteasome inhibitor MG132 suppressed agonist R837-induced TRIM65 degradation, instead of autophagy inhibitor 3-Methyladenine (3-MA), suggesting that the reduction of TRIM65 was due to protein degradation but not autophagy (**Figure 4C**). Meanwhile, the interactions between TRIM65 and NLRP3 during the activation of NLRP3 inflammasome were investigated. PMA-differentiated and LPS priming THP-1 cells were stimulation with the NLRP3 agonist MSU, and then, an immunoprecipitation assay was performed. The results showed that there was a large number of NLRP3 interacting with TRIM65 in inactive THP-1 cells, but this quantity seriously decreased under stimulation with MSU (**Figure 4D**). These results implied that the reduction of TRIM65 weakened the interaction between NLRP3 and TRIM65, and further attenuated the ubiquitination of NLRP3. Next, the effects of TRIM65 on the assembly of NLRP3 inflammasome were assessed. During NLRP3 inflammasome activation, the interaction between NEK7 and NLRP3 is an essential and critical step for the subsequent NLRP3 oligomerization, assembly and caspase-1 activation (32–34). Then, whether TRIM65 could inhibit nigericin-induced the interaction between NEK7 and NLRP3 was examined in BMDMs. The results showed that TRIM65 deficiency remarkably strengthened ATP and nigericin induced the interaction between NEK7 and NLRP3 (**Figure 4E**). Furthermore, whether TRIM65 affected the direct interactions between NLRP3 and NEK7 was also detected. As shown in **Figure 4F**, Flag-NEK7, mcherry-NLRP3 and different dose of GFP-TRIM65 were co-expression in HEK 293T cells. Co-immunoprecipitation assay showed that the direct NLRP3-NEK7 interaction were destroyed by TRIM65. Therefore, these data implied that the reduction of TRIM65 induced by NLRP3 agonists decreased the interaction between TRIM65 and NLRP3, which attenuated NLRP3 ubiquitination and enhanced its interaction with the downstream NEK7, ultimately favoring NLRP3 inflammasome assembly and activation.

### TRIM65 Prevents Multiple NLRP3-Dependent Acute Inflammation *In Vivo*

**Figure 5:**
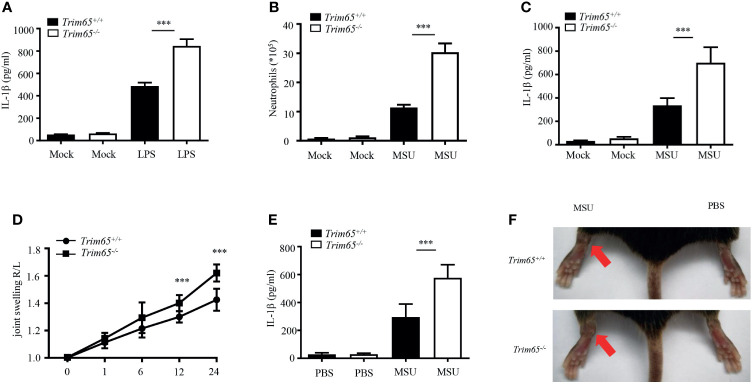
TRIM65 signaling prevents LPS-induced systemic inflammation and MSU-induced peritonitis and gouty arthritis *via* suppression of the NLRP3 inflammasome. **(A)** *Wild-type* or *Trim65^-/-^* mice (5 mice per group) were intraperitoneally injected with LPS (15 mg/kg) and treated for 4.5 h. The sera were collected, and the levels of IL-1β were measured by ELISA. **(B, C)** *Wild-type* or *Trim65^-/-^* mice (4-5 mice per group) were intraperitoneally injected with MSU crystals (1 mg/mouse) and treated for 6h. The numbers of neutrophils in the peritoneal cavity were analyzed by FACS **(B)**, and the levels of IL-1β **(C)** were assayed by ELISA. **(D–F)** *Wild-type* or *Trim65^-/-^* mice (7-10 mice per group) were intraperitoneally injected with MSU crystals, after which joint swelling **(D)** at different time points was measured with a Vernier caliper, the levels of IL-1β **(E)** in the supernatants of joint cultures at 24 h were assayed by ELISA, and changes in joint swelling at 24 h were compared **(F)**. Data are shown as the means ± SEM of two or three independent experiments. Student’s *t*-test, ***P < 0.001.

Now that TRIM65 inhibited NLRP3 inflammasome activation *in vitro*, could TRIM65 also suppress inflammatory responses *in vivo*? Excessive NLRP3 inflammasome activation have been found closely related to a variety of human inflammatory diseases. Herein, three models of inflammatory diseases related to excessive NLRP3 inflammasome activation were used to clarify the function of TRIM65 *in vivo*. The effects of TRIM65 deficiency on LPS-induced sepsis were explored, and the outcomes showed that LPS increased IL-1β production in the sera of TRIM65-deficient mice (**Figure 5A**). Based on the findings of a previous study showing that an MSU-induced model of peritonitis is dependent on NLRP3 inflammasome activation (35), the levels of inflammatory indicators of peritonitis: peritoneal neutrophils and IL-1β were analyzed. As expected, MSU increased IL-1β secretion and neutrophil migration into the ascites of *Trim65^-/-^* mice (**Figures 5B, C**). Multiple studies have shown that the deposition of MSU is the major cause of gouty arthritis, and intra-articular injection of MSU in mice led to NLRP3 inflammasome-dependent arthritis (36, 37). Consistent with previous conclusions, intra-articular injection of MSU caused acute joint swelling and NLRP3-dependent IL-1β production in joint tissue, effects that were strengthened by TRIM65 deficiency (**Figures 5D–F**). Collectively, these findings show that TRIM65 decreased disease severity in multiple mouse models of NLRP3-dependent acute inflammatory diseases (LPS-induced systemic inflammation and MSU-induced peritonitis and gouty arthritis) *via* suppression of the NLRP3 inflammasome.

## Discussion

**Figure 6:**
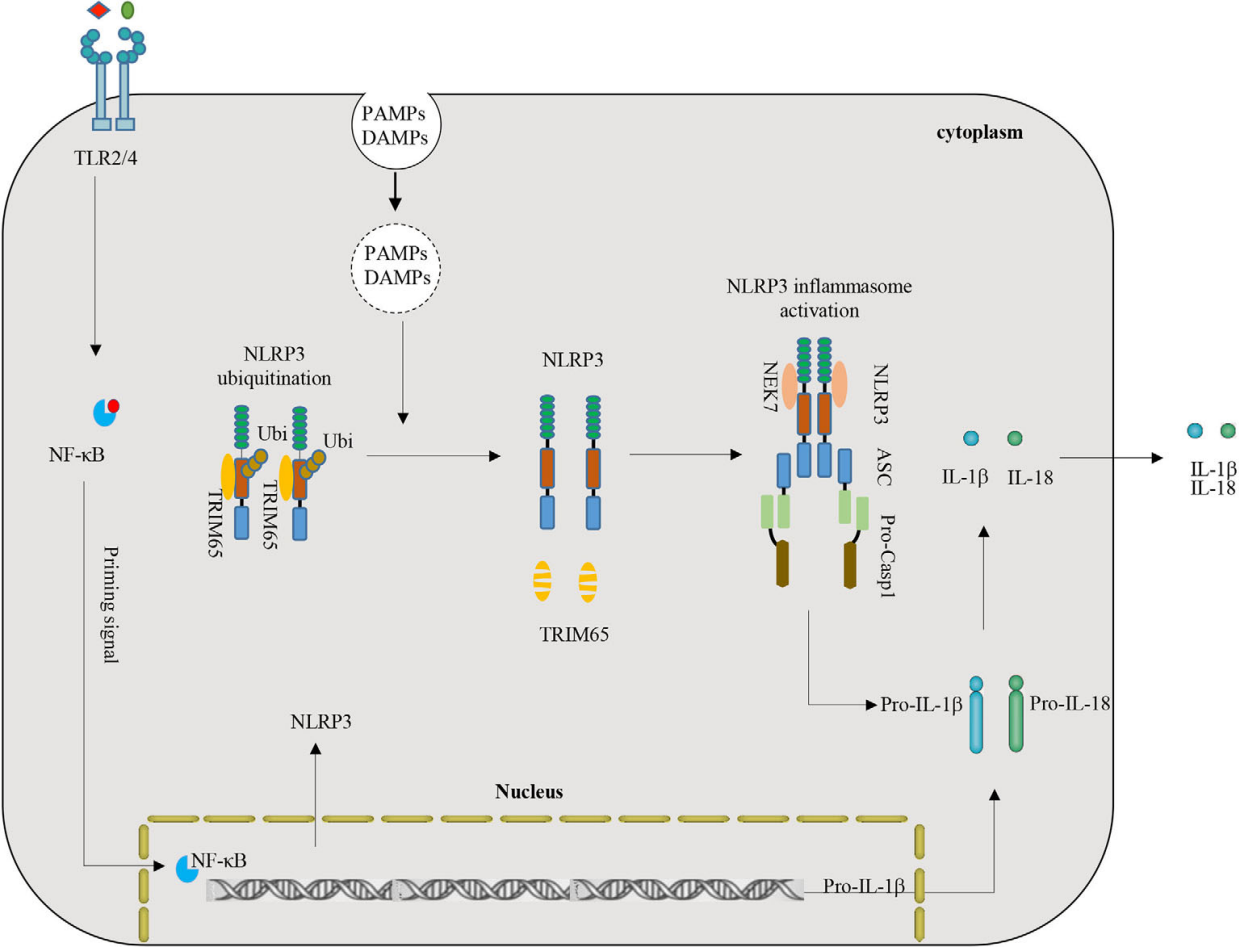
Model of the function and mechanism of TRIM65-mediated inhibition of NLRP3 inflammasome activation. TRIM65 directly binds NLRP3 and promotes K48- and K63- linked ubiquitination, which is critical for the inhibition of NLRP3 inflammasome activation in resting and priming macrophages. With stimulation of priming signals (for example LPS-TLR4-Myd88-NF-κB) and PAMPs or DAMPs (such as MSU, ATP, R837, Nigericin, etc.), TRIM65 significantly decreased, follow by attenuated ubiquitination of NLRP3, strengthened NLRP3-NEK7 interaction, assembled and activated NLRP3 inflammasome. (PAMPs, pathogen-associated molecular patterns; DAMPs, danger-associated molecular patterns).

NLRP3 inflammasome activation plays a key role in inflammation and multiple chronic inflammatory diseases (8, 29). NLRP3, an important intracellular molecular sensor, is tightly controlled and regulated by various PTMs. Ubiquitination is a multifunctional PTM that plays a key role in regulating NLRP3-mediated inflammatory immune responses (6, 38). This study demonstrated a critical role of TRIM65 as a new negative regulatory protein mediated ubiquitination of NLRP3. Depletion of TRIM65 expression advanced NLRP3 inflammasome activation and subsequent inflammatory cytokine production. The mechanism is as follows: TRIM65 directly associates with the NACHT domain of NLRP3 and then induces NLRP3 ubiquitination in resting macrophages. Following stimulation with NLRP3 inflammasome agonists, TRIM65 is markedly degraded, the ubiquitination of NLRP3 is attenuated, the NLRP3-NEK7 interaction enhanced, the NLRP3 inflammasome is assembled, caspase-1 is activated, and mature IL-1β is secreted (**Figure 6**). Taken together, the above results confirmed TRIM65 could suppress NLRP3 inflammasome activation *via* lys48- and lys63- linked ubiquitination and alleviate the aberrant inflammatory response downstream.

Our study revealed the specific role of TRIM65 in inhibiting NLRP3 inflammasome activation. Deletion of *Trim65* enhanced NLRP3 inflammasome activation but had no effect on AIM2 or IPAF inflammasome activation. Consistent with this, we found TRIM65 could interact with NLRP3, IPAF and NLRP1, but not with AIM2. Our further studies showed that TRIM65 could not induce the ubiquitination of IPAF or NLRP1 despite the interaction between them (**Figure S6**). This might explain why TRIM65 inhibited NLRP3 inflammasome activation but had no effect on the AIM2 or IPAF inflammasome. Therefore, the ubiquitination and inhibition of NLRP3 by TRIM65 is specific.

Recently, multiple groups have reported that ubiquitination is a critical post-transcriptional modification to regulate NLRP3 inflammasome activation. So far, three different types of NLRP3 ubiquitination-related negative modulation mechanisms have been found: lys48 ubiquitination-mediated proteasomal degradation of NLRP3 (15–17), lys63 ubiquitination-mediated autophagic degradation of NLRP3 (39) and mixed lys63- and lys48- ubiquitination-mediated NLRP3 inactivation (10, 18, 31). Among them, Kawashima et al. found that ARIH2 Ubiquitinated NLRP3 lys48- and lys63- linked ubiquitination but had no effect on its expression (10), which is consistent with the current study showing that TRIM65-induced mixed lys48- and lys63- linked ubiquitination of NLRP3 merely inhibited its activation but did not cause its degradation. In general, Lys48-linked ubiquitination regulates protein degradation, and Lys63-linked ubiquitination mediates signal transduction (11). However, some studies have shown that proteasome inhibitor (MG-132) does not influence ATP- and nigericin-induced inflammasome activation (40, 41). Inhibition of DUB inhibitors and the regulation of ARIH2 on NLRP3 inflammasome activation and IL-1β production occur independently of proteasome activity (31). As well, it has been reported that heterogeneous ubiquitin chains, such as mixed ubiquitin chains played an important role in ubiquitination (42). Thus, it is sensible that TRIM65 regulates the assembly and activation of the NLRP3 inflammasome through NLRP3 ubiquitination independently of NLRP3 degradation.

Although TRIM65 was reported to have a variety of pathophysiological functions in cancer and viral infectious diseases, including the modification of microRNAs, tumor progression, and regulation of antiviral signals, the role of TRIM65 in NLRP3 mediated inflammatory diseases still have little research (19, 22, 24). One recent report found that TRIM65-deficient mice are more sensitive to lipopolysaccharide-induced lung inflammation and death (23), which laterally supports our results. TRIM65 is involved in NLRP3 inflammasome-related diseases by its ubiquitination of NLRP3 and suppression of NLRP3 inflammasome activation. TRIM65-dificiency enhances disease severity in multiple mouse models of NLRP3-dependent acute inflammatory diseases: LPS-induced systemic inflammation and MSU-induced peritonitis and gouty arthritis. These results suggest that TRIM65 is expected to be a new potential therapeutic target for the treatment of various NLRP3-driven complex diseases.

To summarize, we identified that TRIM65 could suppress NLRP3 inflammasome activation by promoting its mixed lys48- and lys63- linked ubiquitination and demonstrated an endogenous mechanism by which NLRP3 inflammasome activation is negatively regulated. NLRP3 agonists promoted TRIM65 reduction and subsequent attenuation of NLRP3 ubiquitination to favor NLRP3-NEK7 interaction, NLRP3 inflammasome assembly and activation. Moreover, we found that TRIM65 could alleviate LPS-induced systemic inflammation, MSU-induced peritoneal inflammation and gouty arthritis *in vivo*. The data reveal a TRIM65-dependent negative regulation mechanism of NLRP3 inflammasome activation and provide an important target and potential therapeutic strategies for the treatment of NLRP3 inflammasome-driven diseases.

## Data Availability Statement

The raw data supporting the conclusions of this article will be made available by the authors, without undue reservation.

## Ethics Statement

The animal study was reviewed and approved by the University of Science and Technology of China.

## Author Contributions

TT, PL, XZ, RW, XF, and MY performed the experiments described in this work. TT analyzed the data. TT and KQ designed the research, wrote the manuscript and supervised the project. All authors contributed to the article and approved the submitted version.

## Funding

This work was supported by Natural Science Foundation of Beijing Municipality (No. 7184209 to TT), National Natural Science Foundation of China (No. 81803213 to TT) and Research Funds of Profession Quota Budget from Beijing Municipal Science and Technology Commission (2018-bjsekyjs and 2019- bjsekyjs).

## Conflict of Interest

The authors declare that the research was conducted in the absence of any commercial or financial relationships that could be construed as a potential conflict of interest.

## Publisher’s Note

All claims expressed in this article are solely those of the authors and do not necessarily represent those of their affiliated organizations, or those of the publisher, the editors and the reviewers. Any product that may be evaluated in this article, or claim that may be made by its manufacturer, is not guaranteed or endorsed by the publisher.
