# Supplementary material for: The E3 Ubiquitin Ligase TRIM65 Negatively Regulates Inflammasome Activation Through Promoting Ubiquitination of NLRP3
Source: Front Immunol. 2021 Aug 26;12:741839. doi: 10.3389/fimmu.2021.741839 (PMC8427430; doi:10.3389/fimmu.2021.741839)
Supplement: Supplementary file 1 [file DataSheet_1.docx]

Supplementary Material


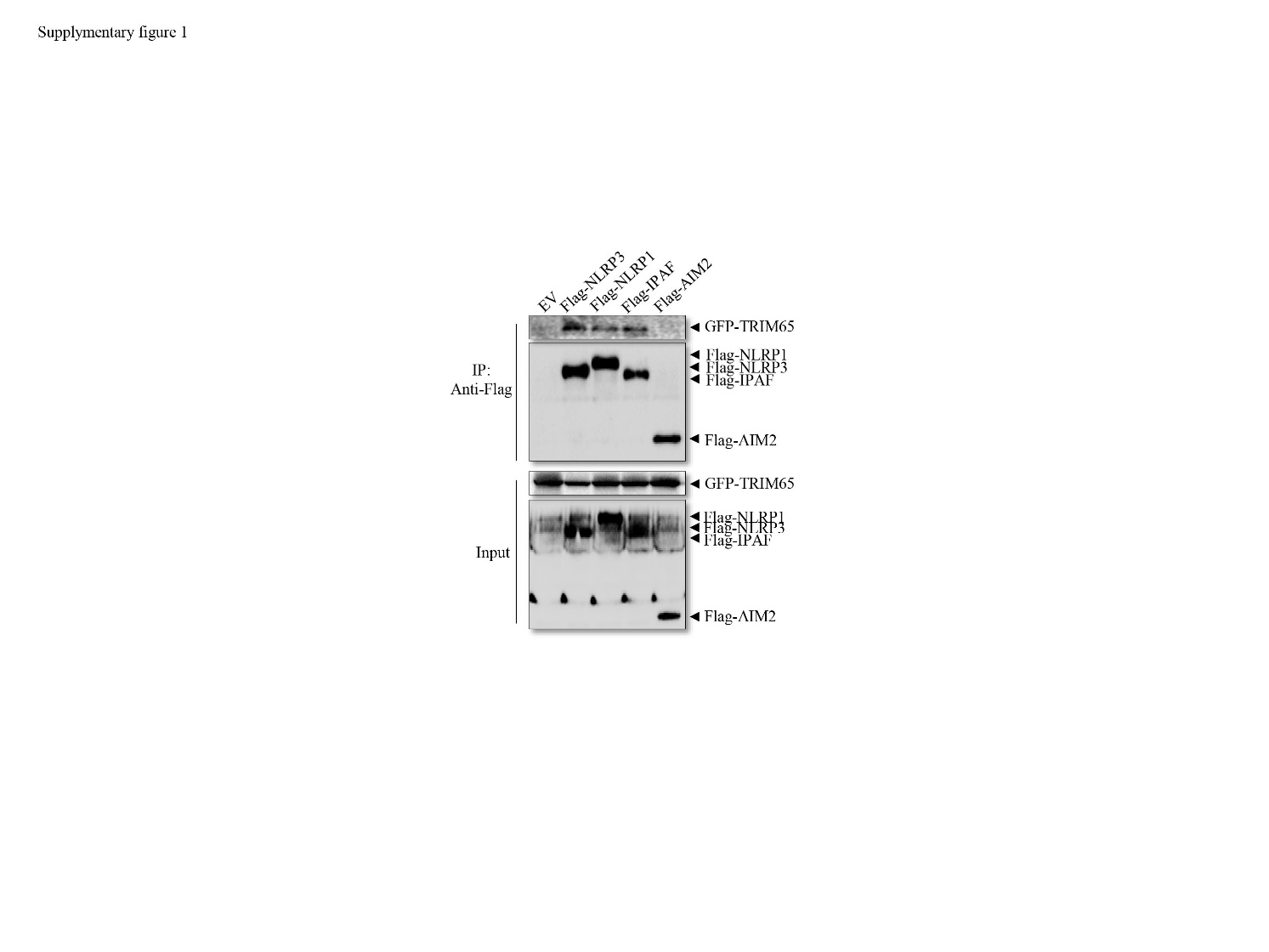


**Supplementary Figure 1. TRIM65 could interact with NLRP1 and IPAF but AIM2.** Flag-NLRP3, Flag-NLRP1, Flag-IPAF and Flag-AIM2 were individually cotransfected with GFP-TRIM65 in HEK-293T cells, and the individual interactions between TRIM65 and NLRP3, NLRP1, IPAF and AIM2 were analyzed by immunoprecipitation and western blotting. EV, empty vector. Data are representative of two independent experiments.

**
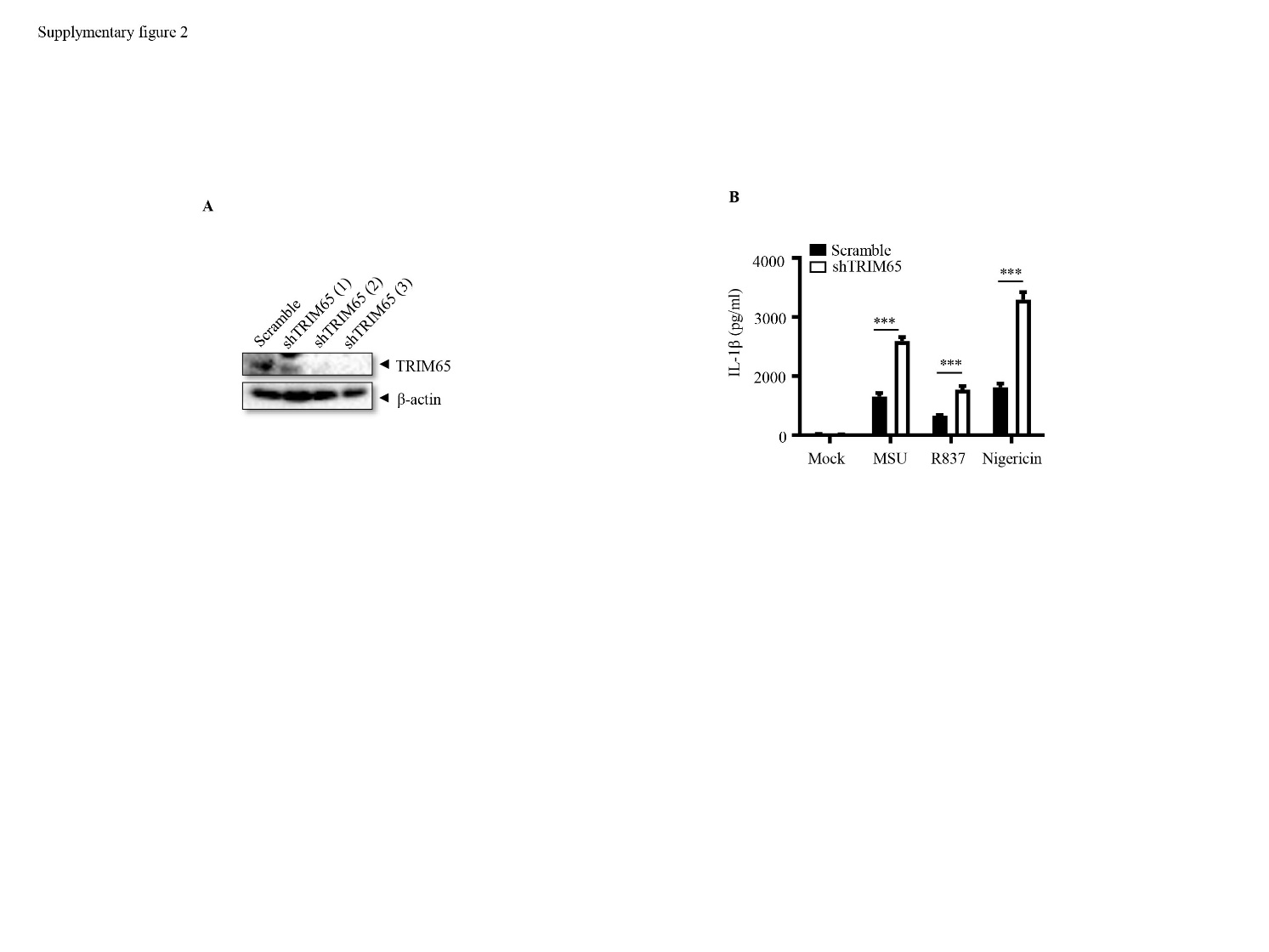
**

**Supplementary Figure 2. TRIM65 inhibits NLRP3 inflammasome activation in THP-1 cells.** (A) Western blotting analysis of the expression of TRIM65 in THP-1 cells stably expressing shRNA targeting TRIM65 mRNA. (B) PMA-differentiated and LPS- primed THP-1 cells stably expressing shRNA targeting TRIM65 mRNA were stimulated with MSU, R837 or nigericin. Supernatants were analyzed by ELISA for IL-1β release. Student’s *t*-test, ****P* < 0.001. Data are shown as means ± SEM of three independent experiments of duplicate biological repeats (B) or are representative of three independent experiments (A). SN, media supernatants; Input, cell extracts.

**
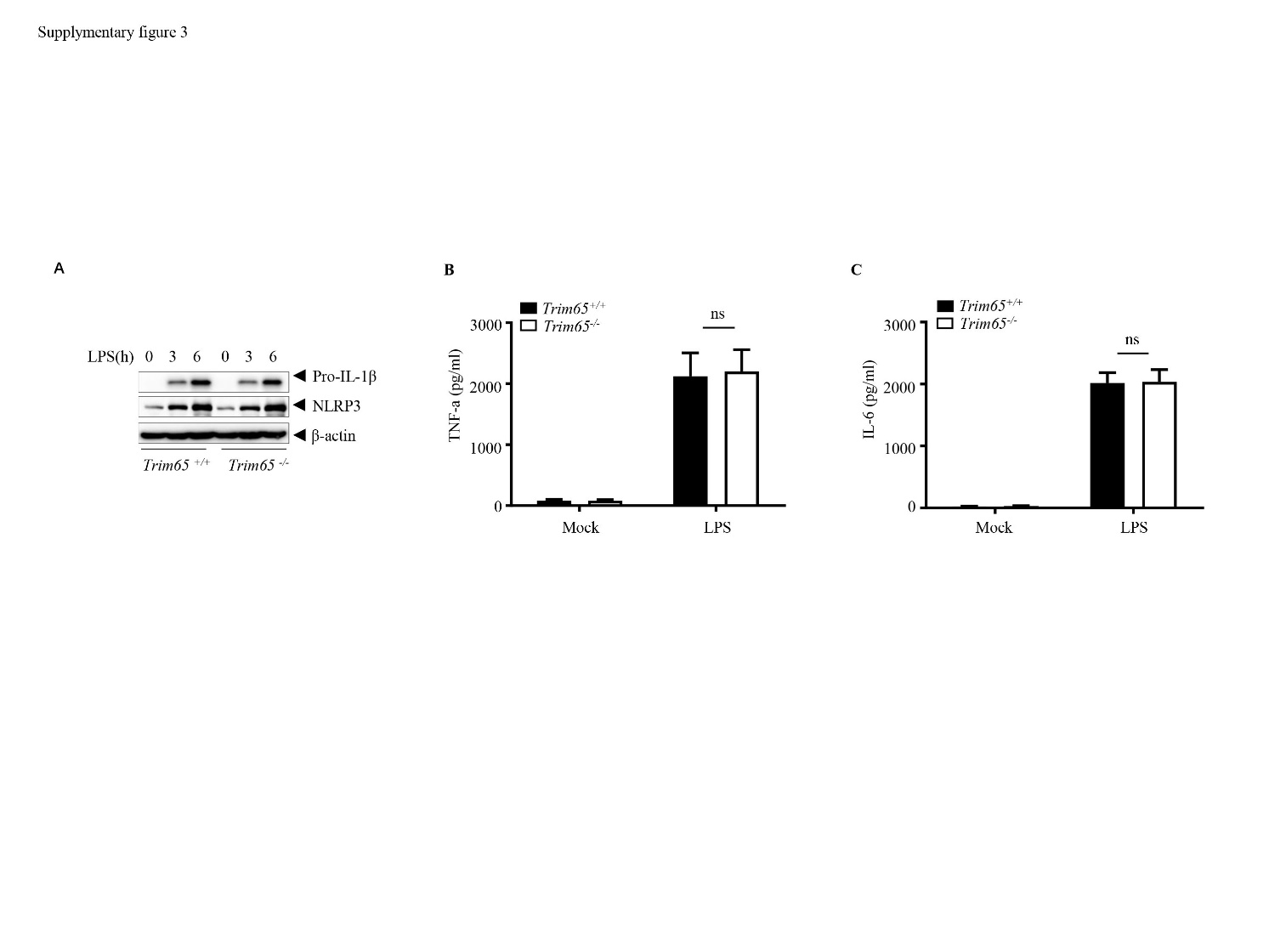
**

**Supplementary Figure 3. TRIM65 has no effect on LPS-induced priming signaling.** (A) BMDMs were primed with LPS for different durations, and the indicated proteins in the cell extracts were analyzed by western blotting. (B-C) BMDMs were primed with LPS for 3 h, and the levels of TNF-α (B) and IL-6 (C) in the culture supernatants were assayed by ELISA. Student’s *t*-test. Data are shown as means ± SEM of three independent experiments of duplicate biological repeats (B, C) or representative of three independent experiments (A). SN, media supernatants; Input, cell extracts.


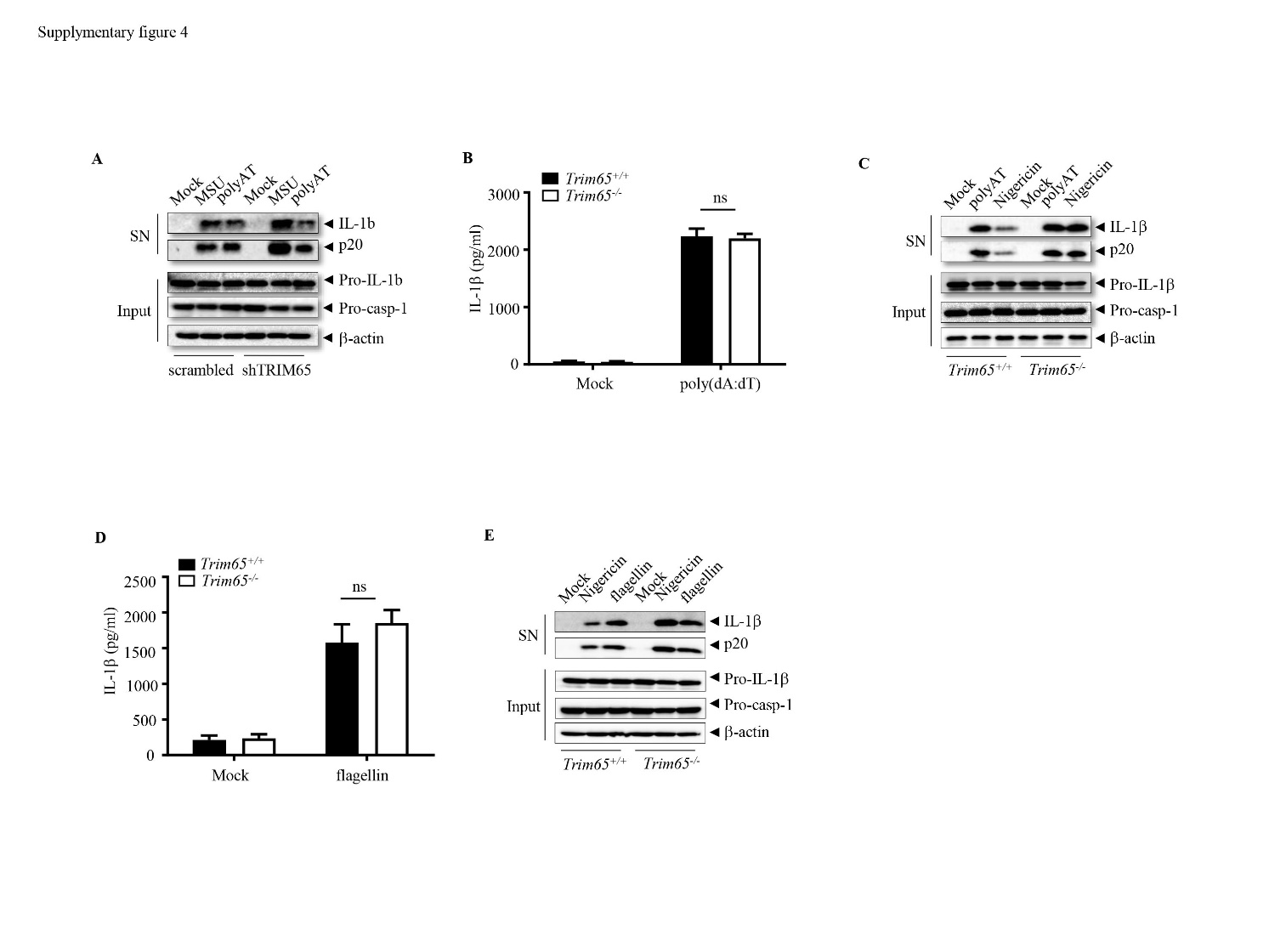


**Supplementary Figure 4. TRIM65 has no effect on AIM2 and IPAF inflammasome activation.** (A) PMA-differentiated and LPS-primed THP-1 cells were stimulated MSU and poly(dA:dT). Cleaved IL-1β, activated caspase-1 (P20) in the culture supernatants and pro-IL-1β and pro-caspase-1 in cell lysates were analyzed by western blotting. (B-E) LPS-primed BMDMs from wild-type and *Trim65^-/-^* mice were stimulated with poly(dA:dT) (B, C) and flagellin (D, E). Cleaved IL-1β in the culture supernatants was assayed by ELISA (B, D). Cleaved IL-1β, activated caspase-1 (P20) in the culture supernatants and pro-IL-1β and pro-caspase-1 in cell lysates were analyzed by western blotting (C,E). Student’s *t*-test. Data are shown as the means ± SEM of duplicate biological repeats of three independent experiments (A, C, E) or representative of three independent experiments (B, D). SN, media supernatants; Input, cell extracts.

**
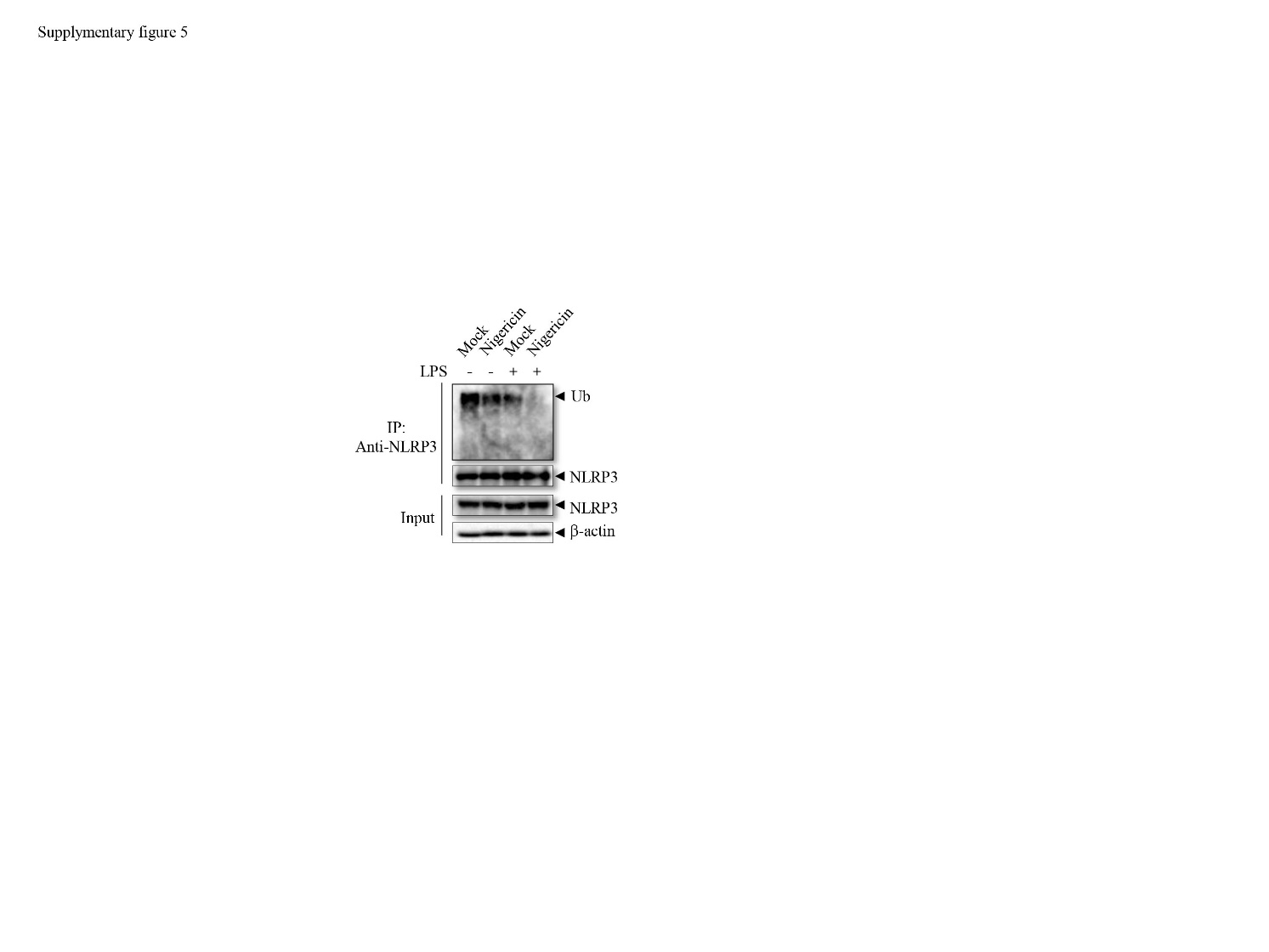
**

**Supplementary Figure 5. Priming and activation signals impair NLRP3 ubiquitination.** PMA-differentiated THP-1 cells were stimulated with LPS, nigericin, or LPS plus nigericin. Ubiquitination of NLRP3 was analyzed by immunoprecipitation and western blotting. Data are representative of two independent experiments.

**
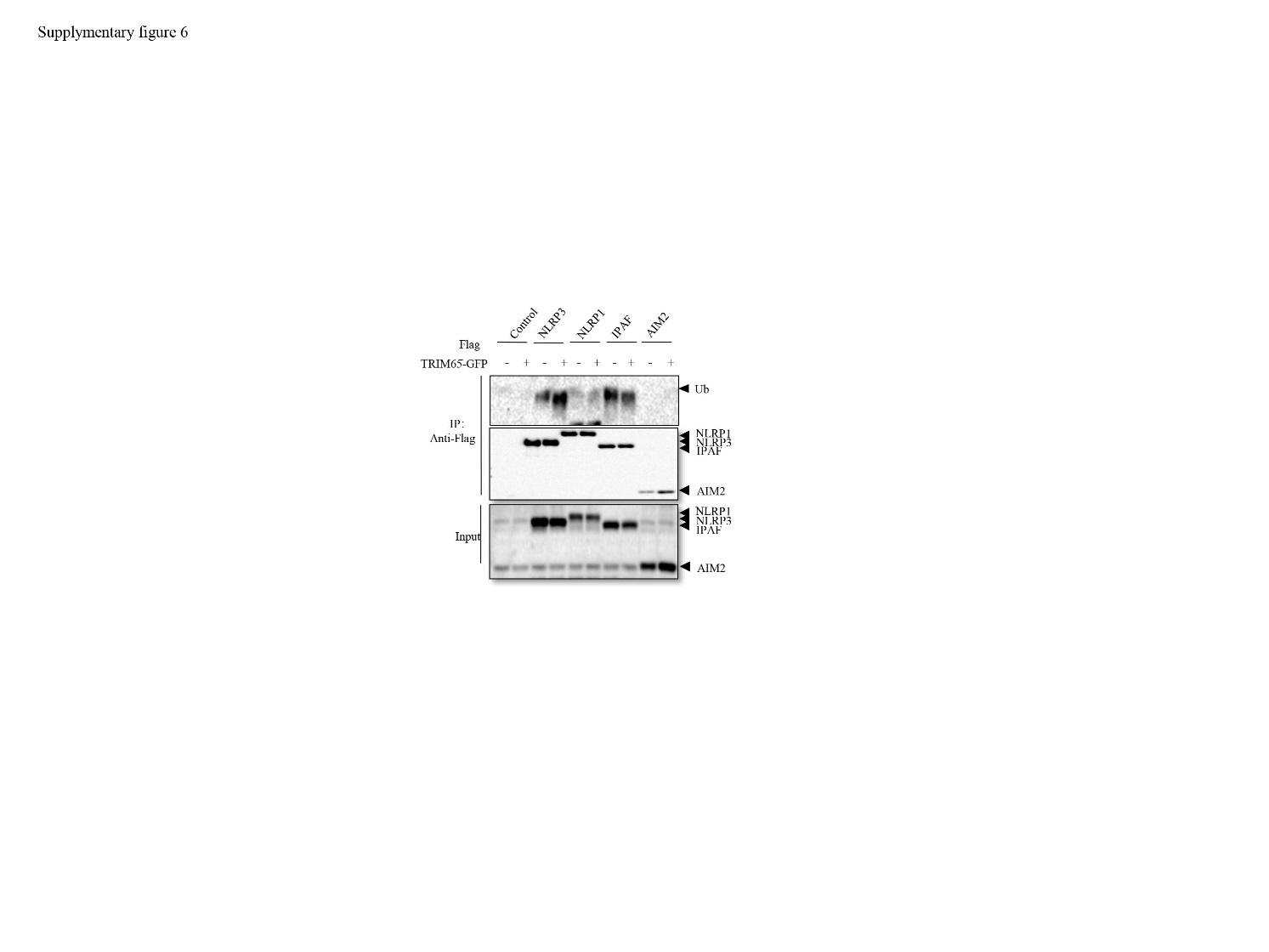
**

**Supplementary Figure 6. TRIM65 could not induce the ubiquitination of IPAF, AIM2 or NLRP1.** Flag-NLRP3, Flag-NLRP1, Flag-IPAF and Flag-AIM2 were individually cotransfected with GFP-TRIM65 in HEK-293T cells, and the ubiquitination of NLRP3, NLRP1, IPAF and AIM2 were analyzed by immunoprecipitation and western blotting. EV, empty vector. Data are representative of two independent experiments.
